# Ambassadors of change: empowering girls from socioeconomically disadvantaged areas through sports and leadership initiatives—an intersectional perspective

**DOI:** 10.3389/fspor.2026.1882523

**Published:** 2026-07-08

**Authors:** Zana Shala, Krister Hertting, Ghazal Zalkat

**Affiliations:** 1 School of Health and Welfare, Halmstad University, Halmstad, Sweden; 2 School of Business, Innovation and Sustainability, Halmstad University, Halmstad, Sweden

**Keywords:** adolescent girls, empowerment, intersectionality, leadership in sports, socioeconomic disadvantage, sport inclusion, youth sports

## Abstract

**Introduction:**

Youth sports in Sweden are socially stratified, reflecting broader societal inequalities. Socioeconomically vulnerable groups, particularly girls from immigrant backgrounds, face significant barriers to participating in sports. Challenges such as gender norms, socioeconomic constraints and culture limit their involvement. Leadership positions in sports remain predominantly occupied by men, further restricting opportunities for female empowerment and representation. Guided by intersectionality as the primary analytical lens and informed by empowerment theory, this qualitative study explores how adolescent girls from socioeconomically disadvantaged areas experience leadership development and community engagement through their roles as ambassadors in a Swedish sport development initiative designed to increase girls' participation and inclusion in sport.

**Methods:**

Using an abductive and interpretive research approach, semi-structured interviews were conducted with ten adolescent ambassadors. The findings identify three interconnected themes: experiencing gender, cultural, and structural barriers; growing into a leader; and empowering the next generation.

**Results:**

The findings demonstrate how girls' participation in sport is shaped by the intersection of gender, socioeconomic disadvantage, migration-related experiences, family expectations, and organizational practices. At the same time, the study shows how community-based leadership initiatives can foster self-confidence, agency, and relational forms of leadership, enabling girls to challenge restrictive norms, negotiate structural barriers, and create more inclusive pathways for others.

**Discussion:**

This study contributes to sport sociology and leadership research by extending intersectional understandings of youth sport beyond mere inclusion, demonstrating how adolescent girls from marginalized communities can act not only as recipients of inclusion initiatives but also as visible leaders and agents of community-based transformation. The findings offer important implications for sport organizations, policymakers, and community initiatives seeking to promote equitable participation, representation, and leadership in youth sport.

## 1 Introduction

“Sport has the power to change the world. It has the power to inspire. It has the power to unite people in a way that little else does. It speaks to youth in a language they understand. Sport can create hope, where once there was only despair. It is more powerful than governments in breaking down racial barriers.”

Nelson Mandela, Laureus World Sports Awards, 2000

Youth sports in Sweden are socially stratified, with societal inequalities also reflected within sports (1, 2). Moreover, disparities in socioeconomic status have increasingly been recognized as critical determinants shaping people's experiences in organized sports (3). Several studies have highlighted how socioeconomic inequality shapes access to and participation in youth sports (3–7). Previous studies on adolescents’ sports participation also indicate that socioeconomic factors play a role in dropout rates from sports (8–10). Furthermore, socioeconomically vulnerable groups are often excluded from sports activities at an early age (5). Strandbu et al. (11) found that girls from minority populations were significantly less active than majority girls, also after controlling for factors such as religion. Similar results were reported by Elling and Knoppers (12), who found that participation among Muslim girls varied by ethnic background, suggesting that factors other than religion itself were more significant.

According to Emmonds et al. (13), there is a significant gender disparity in youth sports participation across Europe, with boys being more active than girls. Foreign-born girls and/or girls with two foreign-born parents have the lowest participation in sports activities (11, 14–16). On the other hand, a Swedish research report found that this group is most likely to participate if given the opportunity (14). A study conducted in Denmark found that adolescent girls from socioeconomically disadvantaged areas face multifaceted and deeply intertwined barriers, where gender, ethnicity, and socioeconomic status intersect (17). Strandbu et al. (11) and van Tubergen and Molteni (16) argue that low participation in sports clubs among girls in lower socioeconomic groups is a consequence of both norms operating within club practices and conventional gender roles that underlie the formation of female identity within these groups. Drawing on a multi-ethnic sample in England, Trigwell et al. (18) identified cross-group similarities in parental understandings of children's physical activity, including limited awareness of guidelines, perceived difficulties in providing support, and the assumption that school-based activity is sufficient. These shared orientations were interpreted as potential constraints on children's activity levels, particularly in contexts where educational attainment is prioritized or within some Muslim communities.

In summary, the proportion of adolescents experiencing multiple and intersecting barriers to leisure participation is highest among girls with foreign backgrounds living in socioeconomically disadvantaged areas. These barriers should not be understood as discrete or independent factors. Rather, gender, ethnicity, and socioeconomic status interact in ways that produce unequal conditions for participation in organized youth sport. An intersectional perspective is therefore necessary to understand how structural inequalities operate simultaneously across multiple social dimensions. Intersectionality, originally conceptualized by Kimberlé Crenshaw (1989), emphasizes that individuals experience marginalization through the interaction of social categories rather than through a single axis of identity. In the context of youth sports, this means that girls from immigrant and low-income backgrounds may face compounded forms of exclusion that are produced by overlapping gender norms, economic constraints, and racialized assumptions about belonging (19–22).

Hence, inclusive access to sport and leisure requires more than formal freedom of choice; it depends on freedom from structural, economic, cultural, and gendered constraints that shape who can realistically participate (23, 24). Consequently, targeted support for clubs operating in underserved areas, together with active outreach and increased accessibility for marginalized groups, is crucial for advancing gender equality and social inclusion in sport (25). Despite existing research on gender disparities in youth sports, few studies examine the intersectional experiences of girls from socioeconomically disadvantaged and immigrant backgrounds in Sweden. Moreover, limited attention has been paid to how leadership initiatives within sports organizations may serve as mechanisms of empowerment under these intersecting structural conditions. Guided by an intersectional framework, this study addresses this gap by exploring how leadership initiatives can foster empowerment and inclusion among girls navigating multiple dimensions of inequality.

## 2 Aim and objective of the study

The following study targets girls who are directly involved in the Tjejsatsningen [The Girls Initiative] project as ambassadors. The ambassadors are girls, often with immigrant backgrounds, who function as facilitators and links between sports clubs and young girls living in these areas. Every participating municipality/area has several ambassadors in special positions, some of whom are both participants and leaders. The ambassadors aspire to be leaders for other girls, share their stories and work on advocacy efforts. They are trained within the project, take part in developing the concept and act as role models for girls in the community.

This study aims to explore how a sports development project in low-SES areas fosters leadership opportunities for young women. By examining participants' perspectives, the study seeks to understand how gendered power relations and structural inequalities shape their experiences in youth sports. Therefore, the objective is to explore the specific barriers these girls face in accessing leadership roles and how they navigate or challenge them.

Specifically, it seeks to answer the following research questions:

1. How do gender, socioeconomic status, and cultural background intersect to influence the leadership development and empowerment of girls in sports?
2. What barriers to sports participation and leadership do the ambassadors identify, and how do they address these challenges?

## 3 Theoretical framework

This study is guided by intersectionality as its primary analytical lens, enabling an examination of how gender, socioeconomic status, and migration background interact to shape adolescent girls' opportunities for participation and leadership in sport. To understand how girls navigate and challenge these structural inequalities, empowerment theory is used to conceptualize leadership development as a process of agency, confidence, and community influence. Finally, research on gender and leadership in sport provides the organizational context through which structural inequalities and opportunities for representation are understood.

### 3.1 Intersectionality as an analytical lens

To understand inequalities in youth sport, gender cannot be examined in isolation from other social and structural positions. Rather, girls' opportunities for participation, belonging, and leadership are shaped by the interaction of multiple dimensions of inequality, including socioeconomic status, migration background, ethnicity, family norms, and local community conditions. The World Health Organization (26) highlights the importance of recognizing the intersecting social determinants that shape experiences of marginalization and health-related disadvantages. To capture this complexity, this study adopts intersectionality as its overarching analytical lens.

Originally conceptualized by Kimberlé Crenshaw (1989) (27), intersectionality explains how systems of oppression such as sexism, racism, and class inequality interact to produce distinct experiences of privilege and marginalization. Crenshaw argues that individuals do not experience inequality through a single social category; rather, exclusion emerges through the simultaneous interaction of multiple social positions. Intersectionality has increasingly informed sport and physical education research, demonstrating how gender, class, race, religion, and migration background interact to shape participation, belonging, and access to leadership opportunities (2, 19–22). Previous studies show that girls from socioeconomically disadvantaged and immigrant backgrounds often encounter multiple and overlapping barriers to sport participation, including financial constraints, parental expectations, gendered cultural norms, spatial inequalities, and institutional exclusion (11, 14, 16, 17). An intersectional perspective shifts analytical attention away from individual deficits and toward the structural conditions that shape opportunities and constraints. In this study, intersectionality enables an examination of how gendered expectations, socioeconomic disadvantage, and migration-related experiences intersect to shape both barriers to participation and access to leadership opportunities in sport.

### 3.2 Empowerment as a process of leadership development and social change

While intersectionality provides a lens for understanding structural inequalities, empowerment theory helps explain how individuals and communities navigate, challenge, and potentially transform these conditions. In line with the United Nations Sustainable Development Goals' gender equality goals, particularly Goal 5, increasing girls' access to participation and leadership is a social and policy priority. Rappaport (28) defines empowerment as a process by which individuals, organizations, and communities gain greater control over decisions that affect their lives. Zimmerman (29) further conceptualizes empowerment across individual, organizational, and community levels, emphasizing that empowerment involves both personal agency and access to structural resources.

The present study primarily focuses on empowerment at the community and organizational levels. Within this perspective, leadership opportunities in sport are understood not only as individual achievements but also as mechanisms through which young women may influence community norms, create social change, and support others facing similar barriers. Previous research suggests that participation in sport can function as an important site of empowerment by fostering self-confidence, leadership competence, communication skills, collective efficacy, and social recognition (30). The empowering potential of sport for girls and women has been both recognized and critically examined for decades (31–35). Participation in leadership roles may enable girls not only to challenge restrictive gender norms, but also to develop agency and legitimacy within their communities. However, empowerment does not occur in a social vacuum. Critical and decolonial sport-for-development scholars have cautioned against universalizing Western notions of empowerment, arguing that such approaches may obscure locally grounded forms of agency, meaning-making, and social change [e.g., (36, 37)]. Hence, access to empowering experiences is shaped by structural conditions such as socioeconomic resources, cultural expectations, institutional support, and organizational practices. Theoretically, this study focuses on empowerment as the enhancement of girls' agency, while also recognizing the crucial process of fostering more cultural awareness and inclusive structures and institutions within organized sports [e.g., (11)]. Empowerment must therefore be understood as both an individual and collective process unfolding within intersecting systems of inequality.

### 3.3 Gender and leadership in organized sports

To understand how empowerment and leadership development occur within organized sport, it is necessary to examine the gendered organizational structures in which these processes take place. In line with the Swedish Sports Confederation's gender equality goals, girls and boys should have equal opportunities to participate in and lead in sport. Despite these ambitions, research consistently shows that women remain underrepresented in leadership positions within sport organizations, particularly in decision-making roles (38, 48, 49). This underrepresentation is even more pronounced among women with immigrant backgrounds, illustrating how leadership opportunities are shaped not only by gender but also by ethnicity, migration history, and social position (39, 40). Research suggests that women's limited access to leadership is closely connected to historically male-dominated organizational cultures, informal networks, and gendered assumptions regarding authority, competence, and belonging (Evans & Pfister, 2021) (41). The absence of women leaders also limits access to mentors and role models, which have been identified as crucial factors for girls' participation, retention, and leadership aspirations within sport (42, 43). Burton (38) therefore argues that leadership in sport must be understood intersectionally, where gender interacts with class, ethnicity, sexuality, and ability to shape opportunities for inclusion and exclusion. Within this study, leadership is understood not merely as formal authority, but as a relational and community-based practice through which young women influence others, negotiate structural barriers, and create pathways for future generations' participation.

### 3.4 Analytical application of the framework

Together, these theoretical perspectives provide an integrated framework for understanding how adolescent girls from socioeconomically disadvantaged areas experience and negotiate participation and leadership in sport. Intersectionality provides the primary analytical lens for examining how structural inequalities shape opportunities and constraints. Empowerment theory explains how leadership roles may foster agency, confidence, and community influence despite these structural barriers. Research on gender and leadership in sport situates these processes within the broader organizational and institutional contexts of organized sport. This integrated framework guides both the interpretation of participants' experiences and the analysis of how leadership initiatives may function as sites of both constraint and transformation.

## 4 Methods

### 4.1 Setting and participants

The study participants were recruited through purposive sampling in order to identify interviewees relevant to the study's aim. The inclusion criteria comprised women and girls participating as ambassadors in the Girls Initiative project. The Girls Initiative is an inclusion program organized by RF-SISU Västra Götaland, one of 21 regional organizations within the Swedish Sports Confederation. The initiative seeks to strengthen opportunities for girls living in socioeconomically disadvantaged areas to participate and remain in organized sport. Through increased access and support, the project aims to enhance girls' independence and participation within sports clubs and to reduce gender disparities in physical activity and sport participation (44). In total, 10 ambassadors agreed to be interviewed, and the participants were aged 16–20 (see Table 1).

**Table 1:** Demographics of participants.

Participant	Age	Sport	Migrant background	Country of birth	Religion	Time in project
TA1	20	Football, handball	Non-European	Non-European	Christian	3 years
TA2	16	Football, basketball	Syria	Syria	Muslim	<1 year
TA3	18	Figure skating, basketball, tennis	Syria	Syria	Muslim	1 year
TA4	17	Basketball	Somalia	Somalia	Muslim	<1 year
TA5	16	Swimming, basketball, football	Somalia	Sweden	Muslim	>1 year
TA6	20	Handball	Non-European	Sweden	Christian	<1 year
TA7	18	Football	Eritrea	Sweden	Muslim	>1 year
TA8	18	Football, Boxing	Palestine	Sweden	Muslim	3 years
TA9	17	Basketball, football	Non-European	Sweden	Muslim	<1 year
TA10	17	Football, swimming	Somalia	Somalia	Muslim	<1 year

The sample size was deemed sufficient based on saturation principles. Saturation was assessed through an iterative process of data collection and analysis. Following each interview, the transcripts were reviewed to identify emerging insights relevant to the study's aim. As data collection progressed, recurring patterns and themes became increasingly evident. After the tenth interview, no substantially new categories or themes were identified, and the material largely reinforced previously established patterns. At this stage, conceptual saturation was deemed reached, suggesting that additional interviews were unlikely to yield further analytical insights. All participating ambassadors were actively engaged in sports initiatives within the areas where they resided.

### 4.2 Data collection

Data collection was conducted in January 2024 through semi-structured interviews with ambassadors participating in the Girls Initiative project. Semi-structured interviewing was chosen to encourage participants to articulate their experiences and perspectives in their own terms while allowing the interviewer to explore themes relevant to the study's aim (45). In line with the interview guide, the interviews focused on the participants' roles within the project and as ambassadors, their experiences with leadership development, and the perceived obstacles and opportunities related to participation and leadership. All interviews were conducted digitally. Given the geographical distance between the researcher and participants, digital interviews provided a practical, time-efficient solution for both parties. Conducting the interviews online also enabled participants to choose a setting that made them feel comfortable and secure during the interview. Each interview lasted approximately 30–40 min.

#### 4.2.1 Researcher positionality

The interviews were conducted by the first author as part of a master's thesis project in collaboration with RF-SISU Västra Götaland. The first author had no prior personal relationship with the ambassadors before data collection and was not involved in the implementation or management of the Girls Initiative project. The second author has been collaborating with the management of the Girls Initiative project for a few years and linked the first author to the project. Participants were informed that participation was voluntary and that their responses would be treated confidentially and would not affect their involvement in the project.

The researcher's academic background in health promotion and interest in challenges inherent in youth participation, empowerment, and social inclusion informed the study. Throughout the research process, reflexivity was maintained through continuous reflection on how personal assumptions and theoretical commitments could influence data collection and interpretation. Special attention was paid to avoiding preconceived understandings of empowerment and participation when conducting interviews and analyzing the data.

All interviews were conducted in Swedish, which was the common language between the researcher and participants. Interviews were transcribed verbatim in Swedish before analysis. Quotations included in the manuscript were translated into English by the first author and reviewed by the co-authors to ensure that the original meaning was retained.

### 4.3 Data analysis

The collected data were analyzed using a thematic analysis (46). An abductive approach guided the analysis, meaning that themes were developed iteratively through an interplay between empirical data and existing theoretical frameworks (47). Given the study's intersectional framework, particular analytical attention was paid to how participants described the interaction between gender, socioeconomic conditions, migration background, family expectations, and organizational structures. Rather than coding these dimensions as isolated categories, the analysis focused on how multiple social positions and structural conditions interacted to shape participants' opportunities, barriers, and leadership experiences. This enabled the identification of themes reflecting both individual experiences and broader systems of inequality. While patterns emerged inductively from the data, these were continuously interpreted and refined in relation to previous research and theoretical perspectives on intersectionality, empowerment, health, and participation in youth sports. This process allowed for the integration of both participant-driven insights and theoretical perspectives. All interviews were audio recorded and transcribed verbatim for accurate data representation. The transcripts were read through several times to gain an overall understanding of the data. The text was divided into meaning units and thereafter condensed, abstracted, and coded. Initial codes were generated across the dataset and continuously compared, both within and across interviews, to identify recurring patterns and variations in participants' experiences. Similarities and differences between the codes were then sorted and compared to create tentative categories. These categories were subsequently grouped into preliminary themes that captured broader patterns of meaning across the data.

The tentative themes were reviewed and discussed to shape the final themes. Throughout this process, the researchers repeatedly returned to the original transcripts to assess whether the developing themes adequately reflected participants' accounts. Codes that did not fit the emerging thematic structure were revisited, reassigned, merged with other codes or discarded. Particular attention was paid to identifying contradictory or divergent experiences that could challenge initial interpretations and contribute to a more nuanced understanding of the data.

Several rounds of discussion among the research team were conducted to refine the thematic structure. During these discussions, theme boundaries were reconsidered, overlapping themes were merged, and theme labels were revised to better reflect the underlying meanings in the data. Analytical notes were also maintained throughout the process to document emerging interpretations and analytical decisions, thereby enhancing the transparency and confirmability of the analysis. The final themes were selected for their ability to capture empirically grounded, theoretically relevant patterns within the study's intersectional framework.

## 5 Results

The analysis identified three interconnected themes: experiencing gender, cultural, and structural barriers; growing into a leader; and empowering the next generation. Together, these themes illustrate how adolescent girls from socioeconomically disadvantaged areas navigate intersecting structural inequalities while simultaneously developing leadership capacities and creating new pathways for others to participate. The findings highlight not only the barriers to girls' access to sports but also the transformative potential of leadership initiatives to foster empowerment, representation, and community-based change. Table 2 provides an overview of the themes and their corresponding insights. The themes provide insight into the factors influencing girls' engagement in sports and potential strategies to promote greater participation.

**Table 2:** Overview of themes, sub-themes, and key insights.

Theme	Sub-theme	Key Insights
Experiencing gender, cultural and structural barriers	Intersectionality	Gender, ethnicity and socioeconomic status shape access to and engagement in sports.
	Parental influence	Cultural norms and safety concerns limit participation: parental engagement fosters change.
	Structural challenges	Limited female role models, gender segregation, lack of resources, and financial constraints hinder participation.
Growing into a leader	Personal development	Increased self-confidence, improved communication, and growth in leadership skills.
	Role of mentorship	Mentorship serves as both an empowering tool for others and a source of self-discovery and development for ambassadors.
	Shifting perspectives	Greater awareness of community needs and commitment to social change.
Empowering the next generation	Representation matters	Female role models, especially those who reflect the girls' own backgrounds, are crucial for inspiration and sustained engagement.
	Creating belonging	Emphasis on building trust, community, and a sense of sisterhood among girls.
	Inclusive strategies	Adapted sportswear, trial activities, and direct engagement with families help reduce barriers and foster participation.

Guided by the study's research questions, the analysis explores how gender, socioeconomic status, and cultural background intersect in shaping both barriers to sport participation and opportunities for leadership development. Across all three themes, the ambassadors' narratives reveal how structural constraints and personal agency are continuously negotiated within family, community, and organizational contexts.

### 5.1 Experiencing gender, cultural and structural barriers

The ambassadors' narratives reveal that barriers to girls' participation in sport were rarely experienced as isolated obstacles. Rather, they emerged through the interaction of gendered expectations, cultural norms, socioeconomic constraints, and organizational practices. From an intersectional perspective, girls' access to sport was shaped not only by individual motivation, but by broader social structures defining who could move freely, occupy public spaces, and engage in organized sport.

Cultural expectations surrounding femininity, clothing, and appropriate female behavior were described as particularly influential. Several ambassadors emphasized that resistance to girls' participation was not primarily rooted in religion, but in broader cultural understandings of gender roles and respectability.

Barriers linked to traditions and cultural views on women have been identified, particularly regarding clothing and expectations of how girls should behave. The ambassadors describe how cultural norms often discourage girls from participating in sports as freely as boys, making it one of the biggest barriers to their engagement in physical activity.

“As far as religion is concerned, that doesn't prevent it [participation]. But it’s the culture that stands in the way.” (TA5)

Parents emerged not merely as family members, but as critical gatekeepers of girls' mobility, visibility, and access to public sporting spaces. Parental resistance appeared closely linked to migration-related protective strategies, community norms, and concerns about girls' safety and reputation.

“Culture can be a bit of a clash, and sometimes it can be hard to persuade parents to let their kids start if it’s not something they're used to.” (TA6)

“Many girls are allowed to participate in sports when they are young, but as they grow older, especially during adolescence, they often stop, frequently due to cultural norms and traditions.” (TA3).

The ambassadors' narratives reveal that parental attitudes functioned as a central mechanism through which gendered inequalities in sport participation were maintained. Rather than reflecting isolated family preferences, these attitudes appeared embedded within broader cultural understandings of femininity, respectability, and appropriate female behavior. For girls from immigrant families living in socioeconomically disadvantaged areas, access to sport was therefore shaped not only by individual motivation, but by intersecting family, community, and structural expectations.

Several ambassadors describe how parents, particularly in immigrant communities, often perceive sports as an activity more suited for boys than for girls. This belief results in girls receiving less encouragement, attention and resources to pursue sports. They highlight the need to challenge these traditional views and create more opportunities for girls to engage in physical activities.

“It’s about girls staying at home and cooking while the boys go out.” (TA7)

“It’s very challenging to convince parents to feel comfortable letting their daughters take part in sports. Many still see sports as something that isn't meant for girls.” (TA2)

Parental concerns also play a significant role in limiting girls' participation in sports. Some parents worry about their daughters' safety when traveling to and from practice, particularly in the evenings.

“There are a lot of mothers who are worried and don't want to let their daughters go to practice alone, especially when it’s dark outside. They're afraid something will happen on the way there or home.” (TA4)

Ambassadors describe conversations with parents who are reluctant to let their daughters participate in sports, viewing it as an unsuitable or unnecessary activity.

“I've had conversations with parents where they're very against their daughters playing sports or say they can't because they're ‘doing other things’. It’s a cultural thing and it’s hard to change their view.” (TA3)

This experience was echoed by another participant who explained:

“The parents pulled them back because they were girls.” (TA5)

The ambassadors highlight that parents play a crucial role in enabling or restricting girls' participation in sports. Supportive parents not only encourage their daughters' interest in sports but also create the necessary conditions for them to take part. This influence is particularly significant in relation to cultural norms and values, which can either facilitate or hinder a girl's access to sports. One ambassador explains how parental involvement is essential.

“You want the parents to be involved as well. You want the parents to help, because we can only help so far. Then it ends. Many of them have taboo opinions […] so you have to understand where they come from to be able to give them better information”. (TA1)

At the same time, supportive parents are described as crucial facilitators. Their encouragement, logistical support, and emotional backing enable sustained engagement and counterbalance restrictive norms.

“My friends weren't allowed to play sports: they didn't have that right. The parents pulled that away from them. […] My dad has always supported me in sports. He has been there and helped, he has driven me to practice and competitions.” (TA9)

Another participant described more restrictive expectations:

“Many people around me said that girls shouldn't play football, that it’s okay when you're little but that you should think about your future and study. Today I regret it a lot—why would I stop doing a sport I'm really passionate about?”. (TA10)

Beyond family dynamics, the ambassadors identify broader structural barriers, including financial constraints, limited access to appropriate sportswear, lack of female role models and gender segregation within sports environments. These barriers are described as systemic and therefore more difficult to influence at an individual level.

“For some girls, it’s about not having the right clothes to be able to do sports. We're trying to solve that by finding alternatives and talking to clubs about offering more adapted clothes.” (TA5)

Dress codes were also mentioned as exclusionary for girls wearing hijab, illustrating how institutional regulations may unintentionally reinforce exclusion. The ambassadors' accounts further illustrate how organizational norms, such as standardized dress codes, may unintentionally reproduce exclusion. For girls wearing hijab, participation was not simply a matter of personal choice, but of negotiating institutional expectations that often privilege culturally normative forms of athletic embodiment. These findings suggest that inclusion in sport is shaped not only by access, but by whose bodies and identities are considered legitimate within sporting spaces.

“Some girls who wear a hijab can feel restricted by dress codes, and I think we need to make adjustments to make sports more accessible for all girls.” (TA8)

The ambassadors emphasize how letting parents experience sports through their own activities is a way to break down prejudices and change the view of girls' participation. By providing parents with opportunities to experience and understand the importance of sports, their view of girls participating in sports can change. The key is also to create relationships and build trust with the parents to increase their support.

“We had a bathhouse for just mothers this summer and a lot of people learned to swim. Now they ask us all the time when the next opportunity will be.” (TA5)

The ambassadors further discuss different solutions to deal with these barriers. They mention increased education and information for parents as well as organizing inclusive trial activities with sports clubs to encourage more girls to try sports and strengthen girls' confidence.

“We had an event for mothers and daughters and there the mothers got to try it out… so we aroused their interest there.” (TA7)

Thus, while the barriers were described as deeply rooted, the ambassadors also articulated concrete practices through which resistance could be negotiated.

The findings illustrate that girls' experiences in sports are shaped by intersecting social categories—gender, migration background, socioeconomic status, and cultural norms—which together create compounded barriers to participation. Ambassadors describe how cultural expectations around clothing and behavior, parental attitudes, and safety concerns interact with gender to restrict girls' engagement.

Financial constraints and limited access to appropriate sportswear further exacerbate these barriers, particularly for girls from low-income and immigrant families.

“My mother had to manage everything on her own—the fees, equipment, matches, and tournaments- so it was quite challenging for her. There was also no one available to drive me to training sessions.” (TA1)

The ambassadors also note how systemic factors, such as gender segregation in sports environments and the lack of diverse role models, reinforce exclusion. By acknowledging these intersecting oppressions, the ambassadors' advocacy work addresses not only gender disparities but also broader social and structural factors that limit participation. Targeted strategies, such as involving mothers in sports activities and providing culturally adapted sportswear, reflect an understanding of intersectionality in practice, as these interventions simultaneously address multiple disadvantages.

### 5.2 Growing into a leader

Leadership development emerged not as a formal acquisition of authority, but as a gradual process of identity transformation. Through repeated encounters with parents, peers, and younger girls, the ambassadors developed not only communication skills and confidence, but also a growing sense of legitimacy in occupying public leadership roles, roles many had previously perceived as inaccessible. From an empowerment perspective, these experiences can be understood as a shift from passive participation toward active community agency.

The ambassadors express how their participation in the project has helped them develop self-confidence, communication skills and leadership. Through their role, they have gained the courage to engage with others and advocate for girls' right to participate in sports. One ambassador in particular highlights how she now feels empowered to address parents' concerns directly.

“So right now I dare, but before Tjejsatsningen I didn’t dare to have conversations with people who have a different opinion than me. But now I actually dare to go and talk to parents and ask them, ´Why don’t you want to let your children participate? ´ Maybe I can change their perspective and mindset.” (TA2)

Through these encounters, the ambassadors describe increased self-confidence and communicative competence. Leadership development was not framed as formal training alone, but as something emerging through lived practice and repeated interaction.

“I just sat at home before, now I'm out, meeting people and socializing.” (TA4)

Several ambassadors reflected on changes in their leadership style, describing a shift toward greater empathy, reflexivity, and emotional awareness. Their leadership was relational rather than authoritative. One ambassador reflects on her personal development, noting that she was previously a hot-headed, outgoing person. She has learned to understand others better and developed greater empathy, which also affects how she leads others.

“My leadership is all about being a positive and present role model for the children. I believe it’s important to be a safe person they can turn to if they need help or support.” (TA10)

Another ambassador notes that, through her roles as both a coach and an ambassador, she has grown personally by gaining insight into the challenges girls face in sports and by witnessing the impact of support.

“I have become more determined to push for positive change, and I now also set higher goals for myself. My perspective and thoughts on life, especially in underprivileged areas, has shifted, and I have a clearer picture of what needs to change”. (TA9)

Personal experiences shape their motivation to create change. Some ambassadors recall feeling unsupported or excluded in their own sports journeys, which fuels their commitment to ensuring that future generations of girls have a different experience.

“My own experience as a girl in football, where I wasn’t always supported or appreciated, made me more determined to create change. I don’t want other girls to go through the same frustration and exclusion that I did, especially when it comes to family and society’s attitude toward women’s sports.” (TA7)

The ambassadors express how being able to change attitudes and show that it is possible for girls to participate in sports, regardless of cultural or religious barriers, is something that gives them greater self-confidence and motivation.

“It’s not always easy to convince the parents. Some have very strong opinions about what their daughters should do. But I'm not giving up—I'm trying to find ways to make them understand how important it is”. (TA3)

This theme illustrates how leadership emerged through engagement with structural resistance. The project functioned not only as a sports initiative but as a space for identity formation, engagement and the development of community-based agency.

### 5.3 Empowering the next generation

The ambassadors' commitment to mentoring younger girls suggests that empowerment extended beyond individual development and became relational, collective, and community-based. By positioning themselves as visible role models, the ambassadors challenged dominant assumptions about who can lead, who belongs in sport, and whose aspirations are socially legitimate. Representation emerged as particularly significant for girls from socioeconomically disadvantaged and immigrant backgrounds.

The ambassadors reflect on how girls often do not receive the same support as boys and how this lack of encouragement affects their ability to continue and develop in sports. They emphasize the importance of female role models in providing young girls with representation and a broader understanding of what sports can offer.

“It’s important with representation, to see yourself in certain stages and feel like, ‘Yes, this is possible’.” (TA1)

“It’s important to me to show that girls from all backgrounds can belong in sports, even if they don't often see themselves represented.” (TA3)

Lack of representation is also highlighted as a reason why some girls quit sports at an early age. Seeing someone who shares their background and experiences can make a significant difference in a young girl's decision to stay in sports.

“I didn't see anyone like me when I was a kid, and that’s probably one of the reasons I stopped playing soccer. But if girls today see someone like me who’s there for them, maybe they won't give up.” (TA10)

A significant part of the ambassador's work is to make girls feel comfortable in sports and foster engagement, encouraging them to participate. They have also become important role models for many young girls. One ambassador sees her involvement in the project as an opportunity to provide the support she herself lacked.

“I guess it’s healing one’s own wounds in some way, like being there for others because you didn't have anyone there for yourself”. (TA1)

Another ambassador explains how she views herself as a role model for other girls and emphasizes how her experiences and emotional development have strengthened her as a person. She has gone from being unsure about how to express her feelings to actively using her experiences to help others.

“It means a lot to me… I see a smile on their face, and that means a lot to me.” (TA5)

The ambassadors express how they see themselves as role models especially for young girls from socioeconomically disadvantaged areas. They want to give the girls an opportunity to see someone like themselves as leaders.

“I want to be the person they can turn to if they have questions about sports.” (TA6)

They also reflect on the importance of the project and on creating positive changes for girls in disadvantaged areas through sports. It becomes evident that support and engagement are two key factors in their role as ambassadors. Many emphasize the importance of building a strong community and fostering a sense of belonging among the girls they mentor.

“We want to create a sense of sisterhood, for the girls to feel that we are there for them”. (TA10)

Beyond providing support, the ambassadors express a long-term commitment to their role, highlighting their dedication to being present for the girls over time.

“We're not going anywhere. We will always be there.” (TA5)

For some, taking on the ambassador role has been a deeply personal experience, allowing them to give others what they themselves lacked growing up.

“I am something for these girls, something I didn't get when I was little.” (TA10)

Moreover, the role has contributed to their own personal development, fostering confidence, responsibility and a sense of pride.

“The role as an ambassador has made me more confident and responsible… It has also given me a sense of pride in making a difference for girls around me.” (TA2)

Overall, empowerment was articulated not as an abstract outcome but as an ongoing relational process. Through mentorship, representation, and sustained engagement, the ambassadors contributed to expanding girls' perceived possibilities and negotiating restrictive norms within their communities.

## 6 Discussion

This study explored how adolescent girls from socioeconomically disadvantaged areas experience barriers to participation in sport, develop leadership capacities, and create more inclusive opportunities for younger girls through their roles as ambassadors. Guided by an intersectional lens and informed by empowerment theory, the findings demonstrate that leadership development in sport cannot be understood independently of the broader structural conditions in which girls' participation takes place. Rather, the ambassadors' experiences reveal how gender, socioeconomic disadvantage, migration-related experiences, family expectations, and organizational structures interact to shape both exclusion and opportunity.

The findings highlight the profound impact of the Girls Initiative project on the ambassadors' personal growth, particularly in terms of self-confidence, communication skills and the ability to challenge cultural and societal barriers. The participants' reflections suggest that the project has not only helped them grow as leaders but also empowered them to become active advocates for girls' participation in sports. Their ability to engage with parents and advocate for girls' participation in sports demonstrates the initiative's effectiveness in fostering leadership and empowerment (30). By stepping outside their comfort zones, the ambassadors have learned to navigate difficult conversations and overcome personal hesitations, which can be pivotal in overcoming resistance to change. The findings also reveal a shift in the ambassadors' perspectives on structural inequalities. Their growing commitment to advocating systemic change suggests that the project fosters both individual and community empowerment. By taking on leadership roles, the ambassadors not only support girls' participation in sports but also work to shift community perceptions of gender norms and inclusion.

A central contribution of this study lies in extending intersectional research in sport by demonstrating that barriers to participation are not experienced through gender alone, but through the simultaneous interaction of multiple social positions and structural conditions. Consistent with previous intersectional research in sport and physical education (2, 17, 22), the findings show that girls from socioeconomically disadvantaged and migrant communities often encounter overlapping forms of exclusion related to cultural expectations, financial constraints, neighborhood safety, and limited organizational accessibility. However, the present study extends this literature by showing how these inequalities are actively negotiated at the community level through youth leadership. Rather than positioning girls solely as recipients of inclusion initiatives, the findings illustrate how they become active agents who challenge and transform intersecting structures of inequality within their local sport environments.

Girls from marginalized backgrounds often experience multiple layers of discrimination, making it even more difficult for them to access and engage in sports. The ambassadors' efforts to advocate for these girls highlight the necessity of an intersectional approach that acknowledges the complexity of their lived experiences. These insights align with a large body of research that emphasizes the structural and intersectional nature of barriers to sports participation. Youth sports in Sweden are not isolated from broader societal inequalities: rather, they reflect and reproduce them (2). Socioeconomic disparities, migration background and gender have all been identified as critical determinants that shape young people's ability to access and remain in sports (3, 10, 14, 17). The ambassadors' experiences highlight how these factors intersect in complex ways to constrain participation, especially for girls from socioeconomically disadvantaged and migrant communities.

An important intersectional insight concerns the role of family and community norms in shaping girls' access to sport. Parents emerged not simply as individual decision-makers, but as key gatekeepers whose attitudes reflected broader intersections of gendered expectations, migration-related protective strategies, and concerns about safety, reputation, and belonging. This supports earlier research highlighting parental influence on youth sport participation (18) and further demonstrates how parental decision-making is embedded within broader social and structural contexts (11, 16, 40). Importantly, the ambassadors did not approach these barriers as fixed cultural obstacles. Instead, they developed relational and culturally sensitive strategies, such as involving mothers in sport activities and building trust across generations, illustrating how intersectional awareness can be translated into community-based practice.

The findings also contribute to empowerment research by showing that leadership development emerged not as the acquisition of formal authority, but as a gradual process of identity transformation, agency, and community engagement. In line with empowerment theory (28–30), the ambassadors described increased self-confidence, communication skills, and a growing ability to navigate resistance and advocate for social change. However, the findings extend existing empowerment literature by showing that empowerment in sport is not solely an individual outcome. Rather, empowerment emerged as relational and collective, developed through repeated interactions with parents, peers, and younger girls, and through active engagement with structural resistance. Leadership, therefore, became both personal and political practice through which the ambassadors negotiated social inequalities while creating new possibilities for others.

The ambassadors' leadership journeys also represent an important counter-narrative to the continued underrepresentation of women in sport leadership (38) (Evans & Pfister, 2021; Lofty et al., 2023), particularly women from migrant backgrounds (39). Previous research has highlighted how gendered organizational cultures, informal power structures, and limited access to mentors restrict women's progression into leadership roles (15, 38). The present findings suggest that community-based leadership initiatives may function as alternative pathways into leadership by creating spaces where young women can develop legitimacy, confidence, and public visibility before entering formal organizational structures. This is particularly significant for girls whose identities are often marginalized within mainstream sport environments.

Representation emerged as another critical dimension of leadership and empowerment. Consistent with previous research emphasizing the importance of female role models in sport (40, 42, 43), the ambassadors described how visibility and shared lived experiences influenced younger girls' sense of belonging and possibility. However, the findings suggest that representation must also be understood intersectionally. Visibility alone may not be sufficient if role models do not reflect the racial, cultural, linguistic, or socioeconomic experiences of the girls they aim to support. This aligns with critical sport-for-development scholarship (36, 37), which argues that interventions often focus on transforming girls rather than the socio-economic, cultural, and political norms and structures that constrain their participation and opportunities. Ambassadors who shared similar community backgrounds appeared particularly well-positioned to build trust, challenge exclusionary norms, and create culturally relevant pathways into sport participation.

While intersectionality is often applied to understand exclusion and marginalization, the present findings also demonstrate its value in understanding resistance, agency, and transformation. The ambassadors did not simply navigate intersecting inequalities; they actively created culturally sensitive spaces, negotiated parental resistance, challenged organizational barriers such as restrictive dress codes, and expanded leadership opportunities for younger girls within their communities. In this sense, intersectionality not only helps explain barriers to participation but also reveals how marginalized young women may act as agents of institutional and community change.

Taken together, the findings suggest that increasing girls' participation in sport requires more than improving access alone. Sustainable inclusion requires addressing the intersecting structural, cultural, and organizational conditions that shape participation, while simultaneously creating leadership opportunities that enable girls from marginalized communities to become visible agents of change.

## 7 Conclusion

This study explored how adolescent girls from socioeconomically disadvantaged areas experience barriers to sport participation, develop leadership capacities, and create more inclusive opportunities for younger girls through their roles as ambassadors in the Girls Initiative project. By applying an intersectional lens and drawing on empowerment theory, the findings demonstrate that leadership development in sport cannot be understood independently of the broader structural conditions shaping girls' everyday lives. Rather, girls' opportunities for participation, belonging, and leadership are influenced by the intersection of gender, socioeconomic disadvantage, migration-related experiences, family expectations, and organizational practices.

The study highlights the Girls Initiative project's significant role in fostering leadership development and empowerment among young women in socioeconomically disadvantaged areas. Through their experiences as ambassadors, they not only developed essential leadership skills but also contributed to breaking down cultural, structural, and gender barriers to sports participation. However, the structural challenges that persist point to the need for continued advocacy and systemic change to ensure equal opportunities for girls in sports.

The findings contribute to sport sociology and leadership research by extending intersectional understandings of youth sport participation beyond a focus on exclusion alone. While previous research has primarily highlighted the structural barriers faced by girls from marginalized backgrounds, the present study demonstrates how adolescent girls can also act as agents of resistance, negotiation, and community-based transformation. Through mentorship, advocacy, and culturally sensitive engagement with families and local communities, the ambassadors actively challenged restrictive norms and expanded opportunities for younger girls within their sport environments.

The study further demonstrates that empowerment in sport is not merely an individual outcome, but a relational and collective process shaped through leadership, representation, and sustained community engagement. Visibility, shared lived experiences, and access to relatable role models emerged as particularly important for fostering belonging, confidence, and long-term participation among girls from underrepresented communities.

From a practical perspective, the findings suggest that increasing girls' participation in sport requires more than improving access to activities alone. Sustainable inclusion requires addressing the intersecting structural, cultural, and organizational conditions that shape participation, while simultaneously creating leadership pathways for girls from marginalized communities. Sport organizations, policymakers, and community actors therefore have an important role in supporting initiatives that not only include girls in sport, but position them as visible leaders and active agents of social change.

## 8 Limitations and future research

There are several limitations to this study. Firstly, the study includes only 10 ambassadors from a specific project. These participants represent a strong and meaningful case study of young females navigating leadership in sports within a socioeconomically disadvantaged context. Their experiences provide valuable insights into the opportunities and barriers faced by marginalized groups in organized sports. However, the findings may not be directly generalizable to other contexts, regions, or populations due to the initiative's specific nature. This study also focuses on ambassadors involved in the Girls' Initiative project, a specific initiative with unique structures and goals. Findings may therefore not fully apply to other sports inclusion projects or contexts with different frameworks.

Future research could benefit from comparative studies across multiple initiatives and geographical regions to better understand how different structural and cultural factors influence sports participation and leadership development. While the study identifies systemic barriers, it may not delve deeply into the structural or policy-level changes required to address them, focusing instead on individual experiences. Even though the study adopts an intersectional perspective, it may not fully capture the nuanced interplay among factors such as gender, socioeconomic status, and cultural norms, leading to an oversimplification of complex realities. Addressing these limitations in future research could provide a more comprehensive understanding of the dynamics of sports inclusion and leadership development in disadvantaged areas.

## Data Availability

The raw data supporting the conclusions of this article will be made available by the authors, without undue reservation.
